# Commentary: “Aptamer-functionalized hybrid nanoparticle for the treatment of breast cancer”

**DOI:** 10.29245/2578-2967/2018/1.1103

**Published:** 2017-12-07

**Authors:** Ronise Evans

**Affiliations:** Research Technician Build Infrastructure Leading to Diversity (BUILD) Xavier University of Louisiana 1 Drexel Drive New Orleans, LA 70125, USA

**Keywords:** P-glycoprotein(P-gp), Nanoparticle, Aptamer, Knock-down, SiRNA, Target, Specific

## Abstract

The major theme throughout this paper was solving the problem of multidrug resistance (MDR) in chemotherapeutic remediation for breast cancer patients by an aptamer-labeled hybrid nanoparticle to enhance selective delivery of siRNA into tumor cells and produce an enhanced knock-down of P-glycoprotein (P-gp); which was detected mainly by western blot. The primary focus of this study was to know whether labeling nanoparticles with a cancer cell specific aptamer could enhance selective delivery of siRNA into tumor cells leading to enhanced knock-down of P-glycoprotein or P-gp as compared to non-labeled nanoparticles. The goal is to minimize cancerous gene expression by silencing its mRNA. Target specificity is not only key in completing this goal, but it is also necessary regarding, biodegradability, cytotoxicity and immune response. To accomplish this goal, the design methods had to be meticulous and carefully researched and applied.

## Introduction

Thus far, conventional chemotherapy for cancer has been synonymous with notorious undesired side effects with limited accessibility to specific tumor tissues causing systemic cytotoxicity. In addition to causing undirected cytotoxicity, modern chemotherapy has evidence of cancer metastasis recurrence, creating ample opportunity for multidrug resistance; allowing cells to develop cross resistance to not only a single drug class but other functionally and structurally unrelated drugs. This study explored amphipathic lipid based nanotechnology in coherence with RNA interference to knock-down cancer genes to overcome chemoresistance mechanisms. siRNA as an anticancer therapeutic, depends on the availability of a vehicle that can be systemically administered safely and repeatedly to cancerous cells. It was important in this study that whatever is carrying the siRNA can protect its integrity while permitting efficient release from the vehicle within the cell. The choice of each component for the formulated nanoparticle contributes to its target specificity, constructive integrity or, aid in limiting cytotoxicity and counteractive immune response. It has been proposed that incorporating aptamer complexes into liposomes will improve specific drug loading and offer better control over the release rate to optimize the therapeutic efficiency of chemotherapeutic drugs. Aptamers are a great choice in formulation of a targeted nanoparticle because they can be compared to antibodies in their ability to recognize a specific target due to the unique higher order structure which confers on them the high affinity and specificity for their broad range of target molecules. In comparison to antibodies, aptamers offer lower immunogenicity, increased thermal stability, are readily available for large-scale synthesis and, have lower production costs.

## Body

The idea of this paper was to circumvent MDR by investigating gene knockdown from a siRNA-based nanoparticle which is target specific to HER2+ human breast cancer cells. It was obliging how the paper differentiated the mechanism of MDR not being understood vs. a contributing cause of MDR, being ABC transporters. Aptamer based nanoparticles are best suited to achieve high drug loading while maintaining efficient drug release and therapeutic activity. The hypothesis of this study was that conjugating nanoparticles with cancer cell specific aptamer should allow selective delivery of therapeutic drugs to tumor cells leading to enhanced cellular toxicity and antitumor effects as compared to unconjugated nanoparticles. This can be presumptuous since, although an anticipated goal of future work comprises of drug delivery, this paper did not discuss any form of incorporation of drugs or drug delivery other than the siRNA intervention. If this anticipation is imminent, what is being done in preparation for nanoparticle formulations to incorporate such drugs? In addition, since the liposome appears to aggregate the siRNA and the aptamer in vivo, the efficiency of delivery/uptake should have been compared in with and without the liposome; especially stability as an intact siRNA after entry to the cells invivo.

Five different breast cancer cell lines (human MDA MD-231, MCF-7, SKBR-3, chemo-resistant mouse 4T1-R) were employed for the present study. During the encapsulation of the siRNA in preparation for the aptamer-labeled nanoparticles, there was some uncertainty of what kind of siRNA was encapsulated into which cell line. Although this study and the title of the paper aim to deliver siRNA via this formulated nanoparticle into breast cancer; the title is misleading because it does not stipulate the specimen being tested for breast cancer, when in fact throughout the article, there are several types of cell lines being tested, the title should clearly reflect that as well. There could have been more clarity in the purpose for using so many cell lines. If P-gp is expressed in most to all the cell lines, what is the control? It is understood that the other cell lines were used to show a trend, but could be clearer when explaining what target species this nanoparticle is ultimately for and which cell lines were positive and negative controls. Although aptamers have a great ability to recognize a specific target such as HER-2 receptors on breast cancer cells, siRNA doesn’t essentially need aptamers to gain entry if the siRNA is already specific to the cancer cells. This overall concept can potentially be problematic because cancer cells always over-express RNA or proteins that also present in normal cells. If this is the case, the design may have an astray effect.

## Conclusion

This study explored nanotechnology in coherence with RNA interference to knockdown cancer genes to overcome chemoresistance mechanisms. The results show an increased knockdown of the P-gp gene with lipid-polymer hybrid nanoparticles which effectively and selectively deliver siRNA into the target cells. Although liposomes aren’t a new concept and have a great potential in drug delivery, liposome-based drug formulations have not entered the market in great numbers so far. Some of the problems limiting the manufacture and development of liposomes have been stability issues, batch-to-batch reproducibility, sterilization methods, low drug entrapment, particle size control, production of large batch sizes and, short circulation half-lives of vesicles. In addition, the title and some of the key points may be misleading as the paper focused more on liposome-based nanoparticle rather than the aptamer or drug delivery. Future appliances to this study should consider the application of different amphipathic lipid formulations which include encapsulation of chemotherapeutic drug(s) and siRNA. Going forward, this study should also test for breast cancer genes which are exclusively found in humans or whatever target specimen of interest.
